# Quantifying the Role of Ground Beetles for the Dispersal of *Fusarium* and *Alternaria* Fungi in Agricultural Landscapes

**DOI:** 10.3390/jof7100863

**Published:** 2021-10-14

**Authors:** Nadja Heitmann, Michael Glemnitz, Peter Lentzsch, Ralph Platen, Marina E. H. Müller

**Affiliations:** 1 Leibniz Centre for Agricultural Landscape Research (ZALF), Eberswalder Str. 84, 15374 Müncheberg, Germany; mglemnitz@zalf.de (M.G.); lentzsch@zalf.de (P.L.); ralph.platen@zalf.de (R.P.); mmueller@zalf.de (M.E.H.M.); 2 Department of Ecology, Brandenburg University of Technology, Cottbus-Senftenberg, Platz der Deutschen Einheit 1, 03046 Cottbus, Germany; 3 Berlin-Brandenburg Institute of Advanced Biodiversity Research (BBIB), Altensteinstr. 34, 14195 Berlin, Germany

**Keywords:** insect-vector, plant disease spread, Carabidae, ground-dwelling arthropods, *Fusarium*, *Alternaria*, phytopathogenic fungi, mycobiota, wheat, qPCR

## Abstract

The spread by arthropods (zoochory) is an essential dispersal mechanism for many microorganisms, like plant pathogens. Carabid beetles are very abundant and mobile ground-dwelling insects. However, their role in the dispersal of economically relevant phytopathogens, like *Fusarium* and *Alternaria* fungi is basically unknown. We quantified the total fungal, *Fusarium*, and *Alternaria* load of carabid species collected in the transition zones between small water bodies and wheat fields by screening (i) their body surface for fungal propagules with a culture-dependent method and (ii) their entire bodies for fungal DNA with a qPCR approach. The analysis of entire bodies detects fungal DNA in all carabid beetles but *Alternaria* DNA in 98% of them. We found that 74% of the carabids carried fungal propagules on the body surface, of which only half (49%) carried *Fusarium* propagules. We identified eight *Fusarium* and four *Alternaria* species on the body surface; *F.* *culmorum* was dominant. The fungal, *Fusarium* and *Alternaria*, load differed significantly between the carabid species and was positively affected by the body size and weight of the carabids. Carabid beetles reveal a remarkable potential to disseminate different fungi. Dispersal by ground-dwelling arthropods could affect the spatial-temporal patterns of plant disease and microorganisms in general.

## 1. Introduction

The dispersal of propagules by animals (zoochory) is essential for many plants, fungi, and microorganisms like fruit-bearing trees, multiple salvia species, or mycorrhizal fungi [1,2,3]. Moving vertebrates and invertebrates can thereby shape and connects ecosystems, communities, and populations. The mobile link concept integrates, among other mechanisms, zoochory and the movement behavior of the dispersing individual and empathizes the effects this causes on other species [4].

The spatial-temporal movement of vectors for phytopathogens is increasingly recognized as a crucial component of understanding disease patterns in many cropping systems [5,6,7]. Unraveling the three-way interaction of crop plants, phytopathogenic fungi, and an arthropod vector requires an interdisciplinary approach. Nevertheless, the enhancing effects of arthropod activity on plant pathogens’ load are well studied for several plant diseases, like the laurel wilt disease of avocados (*Persea americana* Mill), or the kernel rot of maize (*Zea mays* L.) or Fusarium head blight (FHB) in wheat (*Triticum aestivum* L.) [8,9,10]. Although these three-way interactions seem to be omnipresent, the knowledge about the involved mechanisms is still sparse, especially the role of non-pest arthropods.

Besides fungivory, fungal propagules like spores are ingested (endozoochory) accidentally by arthropods while feeding on plant material that is colonized by fungi [11,12]. Predatory arthropods like spiders and centipedes ingest fungal propagules via their contaminated prey animals. Therefore, fungal propagules can be found in the digestive system or the feces of these animals of different trophic levels [5,13]. Furthermore, the propagules can adhere to the exoskeleton (ectozoochory) of the arthropods while they move between infected plant material [14,15]. Moyo et al. [5] found on herbivory and on predatory arthropods phytopathogenic fungi and showed that herbivore species transmitted the pathogen to healthy plants and that their feces are a source for inoculation. This shows that numerous arthropod species can act as vectors for plant diseases.

*Fusarium (F.)* spp. and *Alternaria (Al.)* spp. (Table Abbreviations and Definitions) are phytopathogenic filamentous fungi that cause immense economic losses worldwide when they infect several crop plants, including wheat [16]. Both genera produce mycotoxins that harm humans and livestock, making their management, and therefore their dispersal mechanisms to one of the greatest concerns in agriculture [17,18]. Wind and rain play major roles in the dispersal of the microscopic spores produced by these two pathogens, but both fungi are also often associated with arthropods [19,20,21]. Especially different *Fusarium* species like *F. avenaceum* (Fries.) Sacc*, F. oxysporum* Schlechtendahl emend. Snyder and Hansen, *F. verticillioides* (Sacc) Nirenberg are vectored by various insect species [9,15,22]. Furthermore, *Alternaria* spp. is frequently isolated from arthropods, like the red flour beetle, leaf cutter ants, or mites [23,24,25]. Therefore, arthropods may play an important role in the dispersal of *Alternaria* spp. as well as of many other fungal pathogens.

Additionally, natural vegetation like grasses and arable weeds, plant debris, or organic matter in the soil are alternative hosts for phytopathogenic fungi like *Fusarium* and *Alternaria* and are frequent sources for new infections of crop plants [26,27,28]. Arthropods can not only move frequently between crop plants but also between alternative inoculum sources and crop plants. Therefore, it is possible that arthropods regularly disseminate fungal propagules between different hosts and can therefore affect the disease pattern in the environment.

The ground-dwelling carabid beetles are very likely to get into contact with *Fusarium* spp. and *Alternaria* spp. and other fungi on weeds, plant debris, or crop plants. Their ecology is very diverse and most carabid beetles are very mobile insectivores and considered beneficial for agriculture [29,30,31]. Nevertheless, some species are granivore or food specialists [32,33]. These beetles are very common in agricultural landscapes and are well studied, except for their contribution to the microbial community and their potential to disseminate pathogens [34]. In general, ground-dwelling arthropods are a promising group when investigating supplementary pathways for pathogen vectors since they are very abundant, highly mobile, and move frequently between semi-natural and agricultural habitats.

This study investigates if and how carabids contribute to the dispersal of *Fusarium* spp.*, Alternaria* spp., and other fungi. We wanted to quantify the fungal, and the *Fusarium* and *Alternaria* load (number of propagules or genomes) of different carabid species. Despite species-specific impacts, we searched for more generic traits explaining fungal loads in carabids, like carabid’s body size and weight. Furthermore, we wanted to identify *Fusarium* and *Alternaria* species and their abundances on the body surface of the carabid beetles.

**Hypothesis** **1** **(H1).** *We hypothesize that carabids frequently get in contact with fungi including Fusarium and Alternaria fungi, and hence we expect to find whose DNA or propagules on and in most of the carabid beetles.*

**Hypothesis** **2** **(H2).** *Additionally, we expect in general a high percentage of Fusarium and Alternaria in the total fungal load, and a higher species number and load of Fusarium than Alternaria fungi because many Fusarium species are known to be dispersed by arthropods.*

**Hypothesis** **3** **(H3).** *Furthermore, we expect that certain traits of the carabids, here the body size and body weight, affect the total fungal, Fusarium, and Alternaria load and that these are higher in larger carabid species.*

We collected carabids with pitfall traps in wheat fields close to semi-natural small water bodies (kettle holes). These pond-like habitats are suspected to be a source for phytopathogenic fungi since they provide moisture and alternative hosts plants for phytopathogenic fungi [35,36]. Fungal propagules on the body surface (exogenous) of the carabid beetles were quantified and *Fusarium* spp. and *Alternaria* spp. were identified with a culture-dependent approach. Additionally, exogenous and endogenous fungal, *Fusarium,* and *Alternaria* DNA, from the body surface and the inner body parts of the carabid beetles were quantified using qPCR-based methods. This paper provides the first insights into the role of a common agricultural non-pest insect in the dispersal of devastating plant pathogens.

## 2. Materials and Methods

### 2.1. Study Site

The study site is located in the Lowlands in North Germany, about 90 km north of Berlin in the county Uckermark in the Federal State of Brandenburg, Germany (GPS coordinates of the study area: between 53°23′19″ N 13°35′2″ E and 53°19′2.28″ N 13°51′47.88″ E). Within this region lies the Agricultural Landscape Laboratory Quillow (AgroScapeLab) of the Leibniz Centre for Agricultural Landscape Research [37], where this study was conducted. The climate in this area is subcontinental with 8.6 °C long-term mean annual temperature and average annual precipitation of 564 mm (ZALF field station, Dedelow). The area is formed by glaciations of the Pleistocene and post-glacial processes and represents typical landscapes in Central continental Europe. The Pleistocene processes created a high number of kettle holes, which are small wetlands or ponds surrounded by semi-natural vegetation margins [36]. Kettle holes act as important hot spots for many arthropods, like bees, carabid beetles, or spiders, in this agricultural-dominated area [38,39].

### 2.2. Sampling Design

We examined the fungal community associated with carabid beetles at four kettle holes laying inside winter wheat fields and one in a triticale field. Kettle holes were chosen according to their distances (at least 50 m) to field borders, roads, or other landscape elements, to the size of its water body (min. 25 m^2^), and its margins (dominated by grasses). We collected carabids inside the cereal fields around the five kettle holes within an 8 m radius apart from their vegetation edges. Glass jars (upper diameter: 6.5 cm) were inserted into the ground and used as pitfall traps. The traps were operated without any preservation fluids to avoid the removal or damage of fungal propagules or DNA.

The carabid handling varied slightly with regard to the demands of the two analysis methods for the fungi. For the culture-dependent method, carabids were stored individually at 5 °C in darkness overnight after sampling. After the microbial analyses, the beetles were stored in 70% ethanol and taxonomically determined. Samples to be used for the molecular-biological analysis were stored at −80 °C and weighted before molecular-biological analysis. Traps were operated for 48 h for each analysis, for the culture-dependent analysis between 11 June 2019 and 13 June 2019, and for molecular-biological analysis between 29 April 2019 and 2 May 2019.

**Figure 1:**
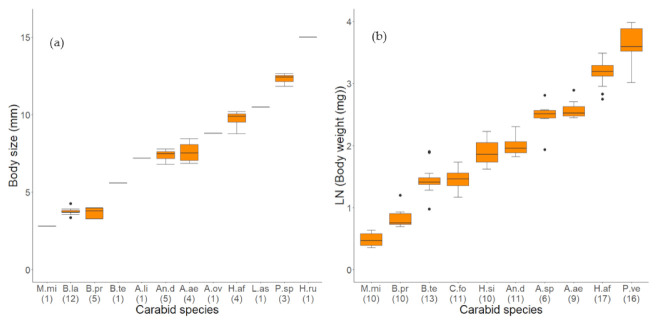
(**a**) Body size in mm, measured from the wingtips to the front of the forehead, of carabid species used to determine fungal loads by a culture-dependent method. (**b**) Body weight in mg (transformed LN(x + 1)) of carabid species, used to determine fungal loading a qPCR approach. Number of individuals per carabid species presented in brackets. Species code according to Table 1 (**a**) and Table 2 (**b**) (abbreviation short).

The taxonomical determination followed the script of Müller-Motzfeld [40] and was conducted for the culture-dependent method after the microbial analyses and for the molecular genetic analysis before. For the culture-dependent method, the body size of the carabid beetles was measured by taking images with a camera (AxioCam ERc5s, Zeiss, Oberkochen, Germany) attached to a stereomicroscope (Stemi 2000-C, Zeiss, Oberkochen, Germany). The body size was measured from the wingtip to the front of the forehead, excluding the mandibles and the last body segment, which can be swollen after the conservation with ethanol, by Zeiss ZEN 2 Blue Software (Zeiss, Oberkochen, Germany). The carabid species and their number of individuals used in the culture-dependent method as well as their body sizes are described in Table 1 and Figure 1a. The carabid species, the number of individuals used in the molecular-biological approach, and their weights are listed in Table 2 and Figure 1b.

### 2.3. Estimation of Exogenous Fungi (Culture-Dependent Method)

#### 2.3.1. Quantity of Fungal Load

We quantified viable exogenous propagules of total fungi and of fungi of the genera *Fusarium* and *Alternaria* on the body surface of carabid beetles. Carabids were kept and dried for 20 min at −20 °C and were then placed individually into 1.5 mL Eppendorf tubes filled with 1.0 mL quarter-strength Ringer’s solution with 0.1% Tween 80, each individual representing one sample. All samples were then placed onto a rotary shaker for 2 min at 30 r.p.m. at room temperature to remove fungal propagules off the body surface via a washing process. The body of the carabid beetles stayed intact to prevent contamination with microbiota from the guts. A total of 0.8 mL of the suspensions were plated onto Petri dishes (diameter 9 cm) with potato dextrose agar (PDA, Carl Roth GmbH Karlsruhe, Germany) supplemented with chloramphenicol (0.4 g/1 L). Petri dishes were incubated for 3 days at 25 °C in darkness followed by 2–4 days under mixed black UV light (emission ca. 310–360 nm) and artificial daylight with a photoperiod of 12:12 h (L:D) at room temperature.

The total of load fungi (TOTAL-CFU/beetle) was calculated by counting all on the nutrient agar germinated propagules as fungal colonies (colony forming units, CFU) and was then extrapolated from 0.8 mL to 1.0 mL washing solution per carabid beetle. The load of *Fusarium* (FUS-CFU/beetle) and *Alternaria* (ALT-CFU/beetle) was calculated by counting these specific CFUs, which were identified on genus level based on colony morphology. After that, the counted CFUs were extrapolated from 0.8 mL to 1.0 mL washing solution per carabid beetle.

#### 2.3.2. Quantity of *Fusarium* and *Alternaria* Species

For a taxonomical identification of *Fusarium* and *Alternaria* species, the fungal colonies were transferred onto a new PDA medium and incubated as described before. *Fusarium* isolates were also placed on Synthetic Nutrient-Poor Agar (SNA; [41]) to develop characteristic micro- and macrospores, chlamydospores, and conidiogenous cells. Identification of *Fusarium* species was based on different micro- and macromorphological features as described by Leslie and Summerell [19] and Yli-Mattila et al. [42]. The identification of *Alternaria* species was based on the microscopic analysis of three-dimensional sporulation patterns after incubation on potato-carrot-agar (PCA; [43]) described by Kahl et al. [44].

### 2.4. Estimation of the Quantity of Endogenous and Exogenous Fungal DNA (qPCR)

#### 2.4.1. Sample Preparation

We quantified fungal DNA from the entire body of carabid beetles, including the body surface (exogenous) and all inner parts (endogenous).

The carabid beetles were weighed individually. The bodyweight of the different carabid species varied immensely between 0.5 mg of an individual of *M. minutulus* and 52.7 mg of an individual of *P. versicolor* (Table 2; Figure 1b). Therefore, a minimal analytical sample weight of 1.0 mg for the less frequent species *M. minutulus* and 5.0 mg for the other carabid species was defined. Up to six individuals of the same species were merged into one sample to reach the minimal analytical sample weight (Table 2). In these samples, the fungal load was averaged for all individuals and finally calculated as DNA genome copy number (gcn) per beetle.

The homogenization of samples was performed in 2.0 mL tubes with a high-speed benchtop homogenizer MP FastPrep 24 (MP Biomedicals Germany GmbH, Eschwege, Germany). Samples were milled at a speed setting of 6.5 m/s for three cycles of 40 s each and stored for 5 min at −80 °C between the cycles. All beetles, except *P. versicolor* and *H. affinis* were milled with two 3.2 mm and one 5.5 mm stainless steel grinding beads (MP Biomedicals Germany GmbH, Eschwege, Germany). *P. versicolor* and *H. affinis* were milled with two 5.5 mm beads in two cycles of 40 s each, followed by another cycle of 40 s with two 3.2 mm and two 5.5 mm beads. To achieve an approximately equal particle size of <0.07 mm for all carabid species after milling, the size and the number of beads, as well as the number of milling cycles for all carabid species investigated, were determined in preliminary tests. Example photographs of milled carabids are given in Figure S1. The particle size of the different beetle species was verified with a camera (AxioCam ERc5s, Zeiss, Oberkochen, Germany) attached to a stereomicroscope (Stemi 2000-C, Zeiss, Oberkochen, Germany) and measured by Zeiss ZEN 2 Blue Software (Zeiss, Oberkochen, Germany).

#### 2.4.2. Genomic DNA Extraction and qPCR

Genomic DNA extraction from the milled carabid beetles followed the standard protocol of the NucleoSpin soil kit, which is specially designed to isolate DNA from microorganisms (Macherey-Nagel GmbH and Co. KG, Düren, Germany). DNA concentration and DNA quality (ratio of absorbance of nucleic acids at 260 nm/280 nm, A_260/280_) were assessed using a NanoDrop 1000 microvolume spectrophotometer following the NanoDrop ND-1000 standard protocol (Kisker Biotech GmbH and Co. KG, Steinfurt, Germany). Therefore, only samples with an A_260/280_ between 1.6 and 2.1 were approved for further analysis with qPCR (Table 3).

Total fungal DNA genome copy number (ITSgcn/beetle) was amplified using the highly conserved rRNA gene primers ITS1F and ITS4, which were specifically designed for basidiomycetes and other fungi [45,46]. The total fungal DNA in a sample was quantified by SYBR green fluorescence qPCR (QuantStudio 12 K flex, Applied Biosystems) using 5.0 μL of template DNA in a 20.0 μL reaction mix (qPCR HRM-mix, Solis BioDyne, Tartu, Estonia). The PCR thermal protocol consisted of an initial 15 min denaturation step at 95 °C, 32 amplification cycles of 95 °C for 30 s, 55 °C for 30 s, 72 °C for 60 s, and a final extension step of 72 °C for 10 min. The quantification of DNA genome copy numbers of *Fusarium* (FUSgcn/beetle) and *Alternaria* (ALTgcn/beetle) by a qPCR approach with genus-specific primers was described in detail by Müller et al. [47]. All qPCR assays contained negative controls and all measurements were performed in duplicate.

### 2.5. Statistical Analysis

All statistical analyses were performed in R 3.6.3. [48]. False discovery rate *p*-value adjustments, based on the Bonferroni-Holm method (a = 0.05) were applied for multiple testing using the package “FSA”. Non-parametric methods for data analysis were used in both methodological approaches according to Zar [49] because the assumptions of homogeneity of variances and normality (tested with the Shapiro–Wilk normality test) were not met in both data sets. The frequency was determined as the number of samples propagules or DNA occurred in and the load as the number of detected propagules or genomes per sample.

To evaluate the relationships of the size or weight, respectively, of the carabids with the fungal load, Spearman correlation matrixes were created, using the package “Hmisc”. A Kruskal-Wallis test compared the differences of fungal load between carabid species in both methodological approaches, followed by a Dunn Test for multiple comparisons of the carabid species. A logarithmic transformation LN(x + 1) was applied to the data of body weight and fungal load calculated in the molecular biological approach. Data for fungal load, DNA concentration, and DNA quality per beetle were calculated by dividing the values for the sample by the number of individuals in that sample.

The midline of all boxplots represents the median, with the upper and lower limits of the box being the third and first quartile, respectively. Whiskers will extend up to 1.5 times the interquartile range from the top/bottom of the box; data beyond that distance (outliers) are represented individually as points. Different letters above the boxplots indicate significant differences between the cara-bid species (Dunn Test adjusted *p*-values, 0.05).

## 3. Results

The main results are summarized in Table 4.

### 3.1. Exogenous Fungi (Culture-Dependent Method)

#### 3.1.1. Quantity of Fungal Load on Different Carabid Species

On the body surface of 29 out of 39 carabid beetles, fungal propagules were detected using the culture-dependent method. The analyzed carabids belonged in total to eight genera and eleven different species (Table 1). In total, 674.4 CFU adhered to the 39 carabid beetles with a median load of 3.6 (IQR 0.0) TOTAL-CFU/beetle. The highest number of fungal propagules (123.0 TOTAL-CFU/beetle) adhered to a single individual of the species *B. tetracolum*. In eleven individuals of four different carabid species, no fungal propagule was found: one *An. dorsalis*, the single individual of *L. assimilis*, five individuals of *B. lampros*, and four of *B. properans*. The last two species are considerably small and morphologically very similar since they belong to the same sub-genus, *Metallina* (Table 1; Figure 1a).

Furthermore, fungi of the genera *Mucor*, *Rhizopus*, *Cladosporium*, *Penicillium*, and *Aspergillus* were identified very frequently on the body of the carabid beetles, but also fungi of the genera *Trichoderma*, *Verticillium*, *Aureobasidium*, *Colletotrichum*, and *Stachybotrys*, as well as different yeasts, were detected.

**Figure 2:**
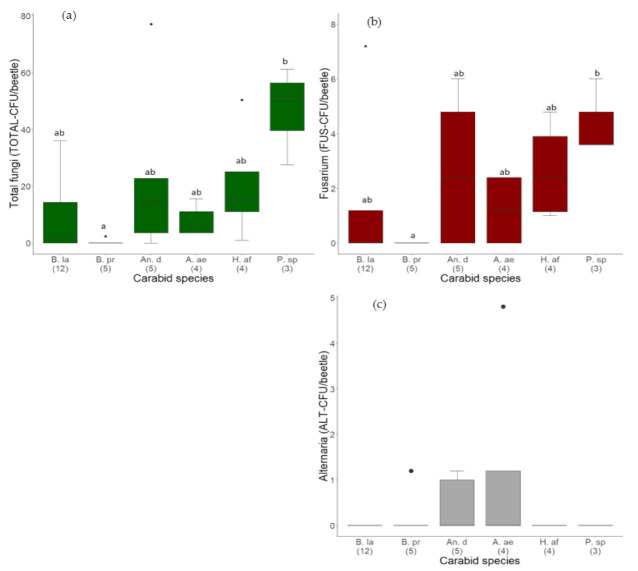
Load of (**a**) Total fungi (TOTAL-CFU/beetle), (**b**) *Fusarium* fungi (FUS-CFU), and (**c**) *Alternaria* fungi (ALT-CFU), detected on the body of carabid beetles with a culture-dependent method. Number of individuals per beetle species presented in brackets. Carabid species are sorted from lightest to the heaviest (median body weight). Species code according to Table 1 (abbreviations short). Black dots in the boxplots are outliers or only one observation per carabid species in (**b**,**c**).

**Figure 3:**
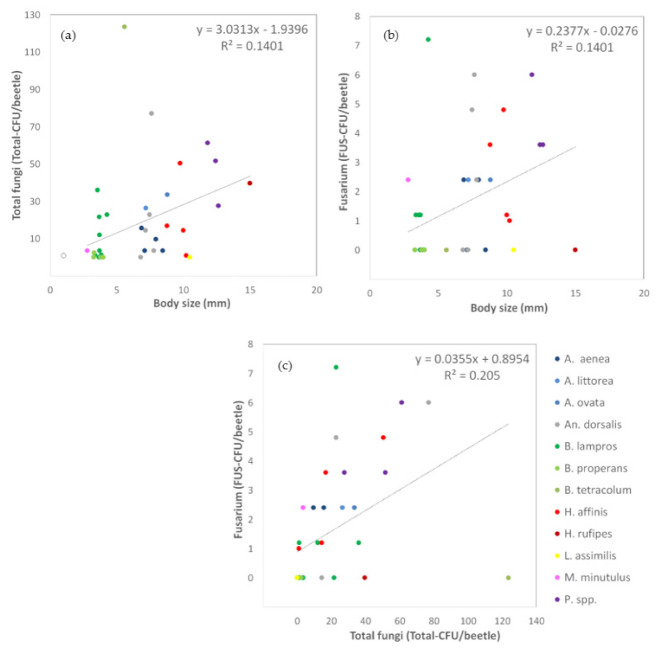
Effects on fungal load (colony forming units, CFU), detected on the body surface of carabid beetles with a culture-dependent method. (**a**) Effect of the body size (mm) of the carabid beetles on the load of total fungi (Total-CFU/beetle), (**b**) Effect of the body size (mm) of the carabid beetles on the load of *Fusarium* (FUS-CFU/beetle), and (**c**) the load of total fungi (Total-CFU/beetle) on the load of *Fusarium* (FUS-CFU/beetle). Carabid species are indicated in different colors in Figure 3c. Species code according to Table 1 (abbreviations long). The dotted lines show a linear trend combined for all carabid beetles.

However, the total fungal load varied significantly between the six most frequent carabid species (H = 14.96, df = 5, *p* = 0.02, N = 33, Table 1). Fungal propagules adhered significantly less to individuals of *B*. *properans* compared to *P.* spp., the largest carabid species in this study (Figure 2a, Table 1). Nevertheless, also individuals of the same species varied immensely in a load of attached fungal propagules. For example, on the body surface of individuals of the species *An. dorsalis*, between 0.0 and 77.0 TOTAL-CFU/beetle were detected. Additionally, the number of fungal propagules adhered to individuals of *H. affinis* varied between 1.0 and 50.4 TOTAL-CFU/beetle (Figure 3a).

**Figure 4:**
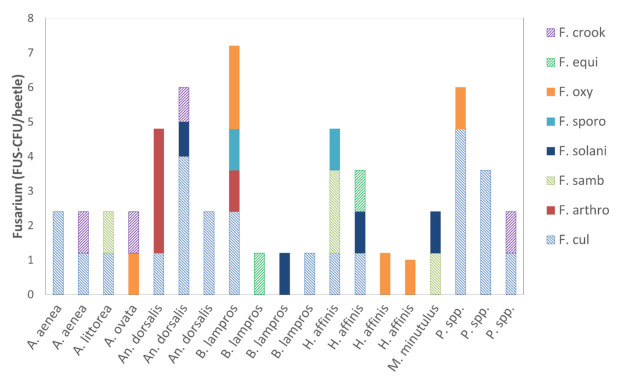
Composition of *Fusarium* (F.) species on the body surface of carabid beetles, detected with a culture-dependent method. Fully colored bars represent *Fusarium* species that produce only macroconidia, striped bars represent *Fusarium* species that produce macro- and microconidia. Species code: *F. crookwellense* (F. crook), *F. equiseti* (F. equi), *F. oxysporum* (F. oxy), F. sporotrichioides (F. sporo), *F. solani* (F. solani), *F. sambucinum* (F. samb), *F. arthrosporioides* (F. arthro), *F. culmorum* (F. cul). For carabid species, abbreviations see Table 1 (abbreviations long).

Propagules of the phytopathogenic and mycotoxigenic genus *Fusarium* adhered to the body surface of 19 out of 39 investigated carabid beetles (Figure 2b; Figure 4). In total, 59.8 CFU were identified as *Fusarium*, representing 8.9% of all detected total fungal CFU (Table 5). In the median, 0.0 (IQR 1.4) FUS-CFU/beetle of *Fusarium* adhered to the carabid beetles. The highest load of *Fusarium* (7.2 FUS-CFU/beetle) was detected on the body surface of an individual of *B. lampros*, the smallest beetle species investigated in this approach (Figure 4). However, the number of attached *Fusarium* propagules varied significantly between the six most frequent carabid species (H = 13.45, df = 5, *p* = 0.02, Table 1). *Fusarium* propagules adhered significantly less to individuals of *B. properans* than to *Poecilus* spp. (Figure 2b).

Furthermore, the frequency and load of the second investigated phytopathogenic genus *Alternaria* were considerably lower than *Fusarium* (median 0.0 (IQR 0.0) ALT-CFU/beetle). Altogether, 9.4 ALT-CFU/beetle (1.4% of total fungi) adhered to the body surface of five out of 39 carabids: two individuals of *An. dorsalis*, one individual each of *B. properans*, *A. littorea*, and *A. aenea* (Figure 2c, Table 5). The highest number of propagules adhered to an *A. aenea* individual with 4.8 ALT-CFU/beetle.

#### 3.1.2. Quantity of *Alternaria* and *Fusarium* Species

Altogether, eight *Fusarium* species adhered to the carabid beetles. *F. culmorum* was detected on 13 carabids and was the most abundant *Fusarium* species (47% of all identified FUS-CFU) (Table 5). *Fusarium* species that produce only macroconidia were more abundant than species that produce macro- and microconidia (Table 5). Four different *Fusarium* species were the highest number detected and were identified on an individual of *B. lampros* (Figure 4).

In total, four *Alternaria* species were identified adhered to the carabids, with *Al. infectoria* being the most common (Table 5). *Alternaria* species that produce high amounts of mycotoxins appeared slightly higher than the ones producing low amounts (Table 5). Propagules of *Fusarium* and *Alternaria* adhered simultaneously to four carabid beetles: two individuals of *A. aenea*, one of *A. littorea,* and one of *An. dorsalis*.

The *Fusarium* and *Alternaria* species were very differently distributed on the beetle’s bodies. For example, on three individuals of the *B. lampros*, three different *Fusarium* species adhered with 1.2 FUS-CFU/beetle each. In contrast, on a further individual of *B. lampros*, propagules of four different *Fusarium* species were attached (Figure 4). Therefore, no obvious association was discovered between *Fusarium* and *Alternaria* species, or between different *Fusarium* species, or between *Fusarium* and carabid species.

#### 3.1.3. Relationships between Carabid Body Size and Number of Fungal Propagules

The number of adhered propagules of total fungi (TOTAL-CFU/beetle) as well as of *Fusarium* (FUS-CFU/beetle) increased with the body size of the carabid beetles. More fungal propagules attached to the body surface of larger beetles, shown by positive moderate correlation coefficients (total fungi: rs = 0.44, *p* = 0.005, N = 39, Figure 3a; *Fusarium*: rs = 0.43, *p* = 0.007, N = 39, Figure 3b). Furthermore, the load total fungal load correlated significantly positively with the number of *Fusarium* propagules on the body surface (rs = 0.71, *p* = 0.000, N = 39, Figure 3c). No significant correlations were found for *Alternaria* propagules since their load and frequency were very low in this approach.)

### 3.2. Quantification of Endogenous and Exogenous Fungal DNA (qPCR)

#### 3.2.1. Comparison of Fungal Contamination between Carabid Species

**Figure 5:**
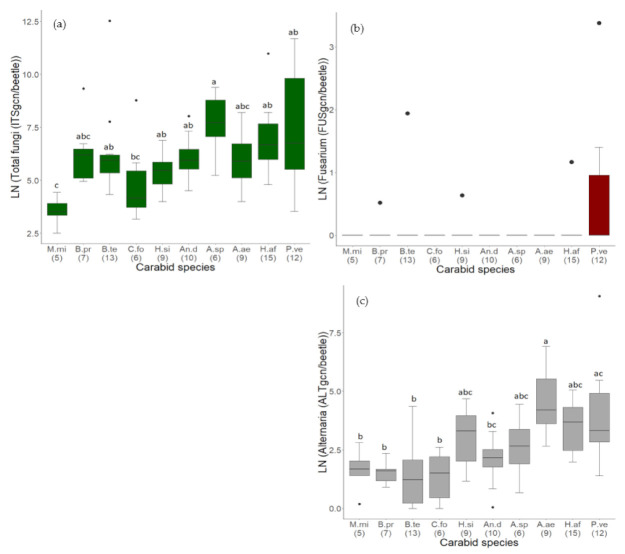
Load of fungal DNA (genome copy numbers, gcn) in samples of carabid beetles comprising the body surface and inner body parts. All data was LN(x + 1)-transformed. (**a**) Total fungi (ITSgcn/beetle), (**b**) *Fusarium* (FUSgcn/beetle) and (**c**) *Alternaria* (ALTgcn/beetle). The number of samples per species is given in brackets and carabid species are sorted from the lightest to the heaviest. Significant differences between species are indicated with a, b, or c. Species code according to Table 2 (abbreviations short). Black dots in the boxplots are outliers or only one observation per carabid species in (**b**).

**Figure 6:**
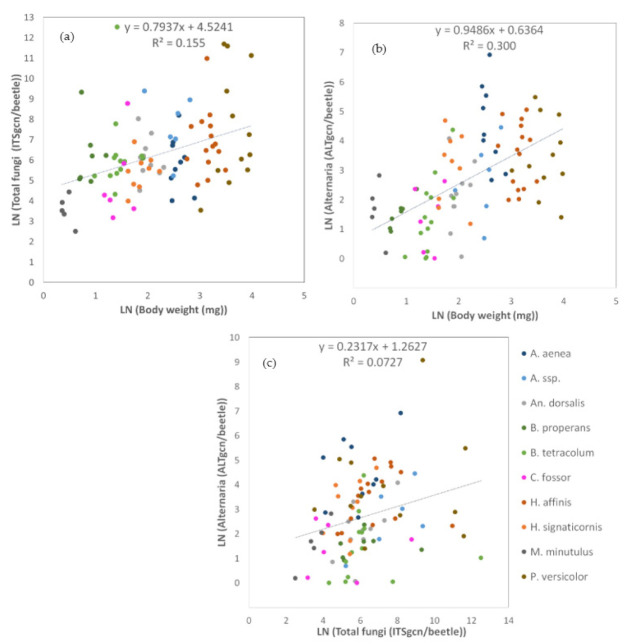
Effects on the load of fungal DNA (genome copy numbers, gcn) in samples of carabid beetles, comprising the body surface and inner body parts. (**a**) Effects of body weight (mg) on the load of total fungi (ITSgcn/beetle). (**b**) Effects of body weight (mg) on the load of *Alternaria* (ALTgcn/beetle). (**c**) Effects of the load of total fungi (ITSgcn/beetle) on the load of *Alternaria* DNA (ALTgcn/beetle). The carabid species are indicated in different colors in Figure c. Species code according to Table 2 (abbreviations long). The dotted lines show a linear trend combined for all carabid beetles.

The total fungal load in 92 samples of 139 carabid individuals belonging to ten different species and seven genera was then analyzed with the qPCR method (Table 3). All samples contained total fungal DNA in very different concentrations with a median of 392.4 and an interquartile range (IQR) of 1102.3 ITSgcn/beetle. The total fungal DNA load varied between 12.3 ITSgcn/beetle in a sample of *M. minutulus* and more than 270,000 ITSgcn/beetle in a sample of *B. tetracolum*. The total fungal DNA load varied significantly between the carabid species (H = 27.587, df = 9, *p* = 0.001, N = 92). In general, a higher fungal load was detected in heavier carabids. The lightest species, *M. minutulus*, showed the lowest values. Their total fungal DNA load was significantly less compared to all other species, except of *B. properans, C. fossor*, and *A. aenea* (Figure 5a), where the differences were not significant. Nevertheless, some species deviated from this generic trend. The total fungal DNA load was significantly higher in *A.* spp. compared to *C. fossor* and *M. minutulus* (Figure 5a). Interestingly, *C. fossor* showed lower levels of fungal DNA, compared to beetles of similar (*B. tetracolum*) or lower weight (*B. properans)* (Table 2, Figure 5a). Furthermore, the total fungal DNA load varied immensely within the same species, especially within *P. versicolor* (from 34.5 up to 119,000 ITSgcn/beetle, Figure 6a). Individuals of other carabid species like *B. tetracolum* and *H. affinis* also showed remarkably high fungal load, marked as outliers (Figure 6a).

Regarding the phytopathogenic genus *Fusarium,* eight out of 92 samples (9%) contained *Fusarium* DNA (Figure 5b). Furthermore, the load of *Fusarium* DNA was overall very low (median 0 (IQR 0) FUSgcn/beetle). *Fusarium* DNA represented 0.006% of the overall detected total fungal DNA. Furthermore, 64% of the *Fusarium* DNA (28.3 FUSgcn/beetle) was found in a single sample of an individual of *P. versicolor*, the heaviest carabid species investigated (Table 2; Figure 5b). Overall, the carabid species *P. versicolor* showed the highest amount of *Fusarium* DNA. Differences between the different carabid species were not significant (H = 13.13, df = 9, *p* = 0.16, N = 92, Figure 5b).

The frequency of the phytopathogenic genus, *Alternaria,* was remarkably higher than that of *Fusarium*. Except for two, all 92 samples (98%) of endogenous and exogenous fungal DNA of carabids contained *Alternaria* DNA. *Alternaria* DNA represented 2% of overall detected total fungal DNA. The overall load of *Alternaria* DNA differed greatly between the samples (median 12.1 (IQR 41.7) ALTgcn/beetle) and ranged from 0.04 ALTgcn/beetle in a sample of *B. tetracolum* up to 8604 ALTgcn/beetle in a sample of *P. versicolor*. Therefore, 67% of all detected *Alternaria* DNA was contained in a single carabid sample. Nevertheless, the endogenous and exogenous load of *Alternaria* DNA varied significantly between the carabid species (H = 40.47, df = 9, *p* < 0.001, N = 92). In general, *Alternaria* DNA was more abundant in heavier species, but *A. aenea* showed the highest load of *Alternaria* DNA. Therefore, *A. aenea* and *P. versicolor* contained significantly more *Alternaria* DNA than the four lightest carabid species (*M. minutulus, B. properans, B. tetracolum, C. fossor*). Additionally, *A. aenea* showed significantly more *Alternaria* DNA than *An. dorsalis* (Figure 5c). In contrast to the total fungal DNA, the four lightest carabid species showed here similar amounts of *Alternaria* DNA (Figure 5c).

#### 3.2.2. Relationship between Carabid Body Weight and the Load of Endogenous and Exogenous Fungal DNA

The results of the qPCR method showed a positive relationship between carabid body weight and the amount of endogenous and exogenous fungal DNA in samples of carabid beetles. The quantity of the total fungal DNA (ITSgcn/beetles) as well as *Alternaria* DNA (ALTgcn/beetle) were significantly correlated with the body weight (ITS: rs = 0.47, *p* < 0.000, N = 92, Figure 6a; *Alternaria*: rs = 0.58, *p* < 0.00, N = 92, Figure 6b). This positive effect of the bodyweight was slightly stronger for *Alternaria* than for total fungi. Furthermore, the load of *Alternaria* DNA and total fungal DNA were significantly positively correlated (rs = 0.36, *p* < 0.000, N = 92, Figure 6b). The moderate correlation coefficient suggests that carabids with a high total fungal DNA load likely contain a high load of *Alternaria* DNA. In contrast, no significant correlations were detected for *Fusarium*.

## 4. Discussion

We explored the loads and frequencies of different fungal taxa, on the body surface and in the entire bodies of different carabid species with a molecular and a culture-dependent method. With a special focus on the phytopathogenic and mycotoxigenic fungal taxa *Fusarium* and *Alternaria*, we identified multiple species of both genera on the body surface of the carabids. We related the total fungal, *Fusarium* and *Alternaria*, load to the body size and weight, and compared the carabid species to identify traits affecting the fungal loads. Interactions between the ground-dwelling carabids and different fungal genera, including *Fusarium* and *Alternaria*, are very frequent in crop fields, are positively affected by the body size and weight, and differ between the carabid species.

The culture-dependent and the qPCR method showed similar trends regarding the total fungal load, but also the differences regarding *Fusarium* and *Alternaria* fungi provided relevant insights. In general, the culture-dependent method quantified only viable and potentially infectious fungal propagules that were washed off the body surface of the carabids. In contrast, exogenous and endogenous fungal DNA of viable or dead propagules was detected together from the body surface and the guts of the carabid beetles in the qPCR approach.

Horizontal transmission of propagules or DNA between individual carabids during the collecting within the pitfall traps could not be excluded with the used methods. Furthermore, in the culture-dependent method, contamination of the body surface with fungal material from the feces or another body secretes is to some extent possible. Both issues should be addressed in further studies in more detail.

The DNA of different fungal genera were detected in 100% of the investigated carabid beetles with the qPCR method. The microbiota in the guts of insects consists of bacteria, protists, archaea, and a few fungi [50,51]. Digestive fungi are common in the digestive tract of insects that feed on detritus or wood, but they probably play a minor role for the investigated carabids since their diet is not wood or detritus-based [50,52]. Furthermore, gut mycobiota are necessary for the immune response and protection against pathogens [53]. In general, the gut mycobiota of insects could either closely relate to the fungi in the food and the environment of the insects, or fungal species composition in the guts could change independently from the environmental mycobiota which suggests a filtering mechanism [11,51]. Commonly found fungal genera in the guts of insects are *Aspergillus*, *Mucor*, *Cladosporium*, *Fusarium*, *Penicillium*, yeasts like basidiomycetes, or ascomycetes [51,54]. The feces of arthropods can be a relevant inoculum source since the propagules of many fungal species stay viable after digestion, like *Fusarium oxysporum*, the Grapevine Trunk Disease Pathogens *Phaeomoniella chlamydospora*, or *Fusarium proliferatum* [5,15,55].

Viable fungal propagules of different genera were detected with the culture-dependent method on the body surface of 74% of the investigated carabids. A considerably lower frequency was detected by plating the appendages and guts of fungivore Collembola [11]. Common fungal genera on insects are *Fusarium*, *Epicoccum*, *Penicillium*, *Aspergillus*, *Cladosporium,* and yeasts like *Candida* [23,56,57]. Fungal propagules of different genera attached very frequently to the body surface of carabid beetles and were possibly transported. In general, propagules can attach to different body parts like the hairs on legs or antennae, or stick to the wings or mouthparts of the insects when the insect is foraging, or moving between infected plant material and get in contact with fruit bodies, mycelia, or spores of the fungi [58,59]. The exogenous acquisition of fungal propagules increases with exposure time to an inoculum source. The transmission of propagule decreases with time after the exposure to the inoculum source [58].

The fungal genera *Fusarium* and *Alternaria* were detected in 49% (propagules, exogenous) and 98% (DNA, exogenous and endogenous), respectively. Higher frequencies for *Fusarium* propagules on the body surface of insects like bark beetles and pigweed weevil (*Hypolixus haerens*) were detected previously [56,57]. However, studies on the banana weevil (*Cosmopolites sordidus*), a known vector for *F. oxysporum*, detected a lower frequency on the body surface [60].

Trunk disease pathogens were isolated from two arthropod species in frequencies similar to the here detected frequencies for *Fusarium* propagules, which were based on these considered effective vectors [5]. Therefore, carabid beetles are probably vectors for *Fusarium* fungi, transporting propagules on the body surface.

Studies investigating the arthropod-mediated dispersal for *Alternaria* fungi, especially studies quantifying endogenous and exogenous fungi separately with molecular methods, are very sparse. With the culture-dependent method, viable propagules of *Al. brassicicola* were often detected in the feces of the flea beetle (*Phyllotreta cruciferae*) and *Al.* spp. were found in the guts of mites [11,59]. On the body surface, viable propagules of *Al. infectoria*, *Al. arborescence*, and *Al. alternata* were recently isolated from Leaf-cutting ants, different collembolan, and the red flour beetle (*Tribolium castaneum*), respectively [11,23,24]. Based on the very frequent detection of *Alternaria* DNA in this study, we suggest that carabids could also be considered a vector for *Alternaria* fungi.

Previous studies showed that *Fusarium* and *Alternaria* propagules stay viable in the feces or the gut of arthropods [15,59]. However, the proportion of viable propagules or the amount of DNA detected in the feces and therefore the actual infection potential should be investigated in further studies. The quantification of transferred fungal propagules and the effect of disease development would be the next step to estimate the relevance of this dispersal mechanism and should be the target of further studies.

Carabids move frequently between semi-natural breeding habitats which are suggested to be a source for phytopathogenic fungi and adjacent crop fields and disperse further into new habitats [61]. Carabid beetles are very mobile and can cover distances of several meters in a random pattern like a correlated random walk and much longer distances in a directed movement pattern, e.g., *P. versicolor* was observed to walk 87 m per day [62,63]. Many species, especially the smaller ones, can fly too or are drifted by wind [34]. These mobile insects can exchange microorganisms and link different habitat types by covering shorter distances very frequently and longer distances from time to time. This movement behavior makes them potentially relevant vectors for several microorganisms. Additionally, carabid species vary in their spatial-temporal load, larger carabid species are usually less common than smaller ones [34]. Therefore, to evaluate the impact of a carabid species on the dissemination of fungi, their species-specific load and movement behavior has to be considered too.

In general, our second hypothesis regarding the higher load of *Fusarium* compared to *Alternaria* fungi was only partly confirmed. We detected frequent and abundant viable *Fusarium* propagules on the body surface of the carabids. *Fusarium* DNA was very rare in the analysis of the entire body and considerably less than *Alternaria* DNA. This is in line with other studies which found *Fusarium* propagules frequently on the body surface of insects, but not in the digestive tract [15,60]. Furthermore, *Fusarium* fungi produce mycotoxins effect for humans and animals [64]. In known insect vectors for different phytopathogenic fungi, reduced survival, fecundity, biomass, and a slower development were observed in the insect species [9,65,66]. These findings suggest that carabids may avoid ingesting food that contains high levels of *Fusarium* and/or its mycotoxins. Nevertheless, carabids beetles and probably other ground-dwelling arthropods move regularly in environments where *Fusarium* fungi are frequent so that propagules can attach to the body surface.

In contrast, *Alternaria* DNA was detected very frequently in the analysis of the entire bodies but viable propagules on the body surface of the carabids were rarely found. In contrast, propagules of *Al. brassicicola* were frequently detected by culture-dependent method on the body surface of flea beetles (*Phyllotreta cruciferae*), and viable propagules were detected in the feces of the flea beetles but this frequency was not given by the authors [59]. *Alternaria* is occasionally detected on the body surface or feces of different arthropods, but seldom in a comparative approach. Nevertheless, the results of this study suggest, that carabid beetles ingest *Alternaria* fungi very frequently and the fungal DNA accumulates in the digestive tract. However, the propagules either do not attach to the body surface of the carabid beetles, or they don’t stay viable as effectively as *Fusarium* propagules since they are more sensitive towards drought stress and UV radiation [67,68].

The fungal load of insects is also affected by the ability of propagules to attach to the body surface, which is mediated by fungal species-specific traits. This includes the spore-bearing structures, physical and chemical properties of the propagules like enzymes, or glycoproteins, or electrostatic recognition systems. Terrestrial spore types can range from dry hydrophobic to sticky hydrophilic conidia [69]. Additionally, our results showed that the production of microconidia did not increase the number of detected CFU and has probably no advantage for the dispersal by the carabids.

Disease-induced plant volatile chemical emissions, caused by a *Fusarium* infection, change the behavior of arthropods. They can be repellent for grain aphids or attractive for sap beetles [66,70]. Carabid beetles also change their behavior according to volatiles send out by plants [71].

In our fieldwork, the data sampling for both methods differed slightly in time, the collection of carabids for the molecular approach were six weeks earlier. We cannot fully exclude that the sampling period and the development state of the vegetation might also affect the fungal community. In further experiments, sampling should be done at the same time.

We identified a complex fungal community on the body surface of the carabids consisting of various *Fusarium* and *Alternaria* species in different frequencies and abundances, including relevant phytopathogens. *F. culmorum* is one of the main agents of Fusarium Head Blight [17] and made up 47% of all detected *Fusarium* CFU. *F. sambucinum*, *F. equiseti*, and *F. sporotrichioides* are regularly associated with FHB and were also detected here in lower frequencies [72,73]. *Al. infectoria* made up half of the detected *Alternaria* CFU on the body surface of the beetles and produces low amounts of mycotoxins [44]. The other three detected *Alternaria* species *Al. alternata*, *Al. arborescence*, and *Al. tenuissima* are pathogens that generate higher levels of mycotoxins and induce diseases like the black point disease, black kernel, and leaf blight [44,74].

In general, arable weeds are an inoculum source for *Fusarium* species, next to different crops like maize and wheat [26]. Most *Fusarium* species can survive on crop residuals, soil, and dead plant matter where they easily interact with ground-dwelling arthropods [75]. *Alternaria* fungi are ubiquitous saprotrophs or opportunistic pathogens and colonize a wide range of plant species, like different types of crops such as small-grain cereals, fruit, and vegetables [76]. Both fungal genera are often found together on wheat plants and compete for the same resources [77].

Competition shapes the microbial community and differences in the saprotrophic capacity of fungal species can affect the species composition [78]. *F. solani*, *F. oxysporum*, *F. poae*, and *F. sporotrichioides* have a better saprotrophic capacity in crop residues or soil than *F. graminearum* [78]. Furthermore, *Fusarium* and *Alternaria* are known antagonists that affect the growth and mycotoxin production of each other [79,80]. Competitive and antagonistic interactions affect the production of primary inoculum and the growth of fungi on the plant residuals and soil and therefore the potential fungal load of insects that share the same habitat [78].

Species-specific traits of the carabid species might also affect the endogenous and exogenous fungal load. Fungal propagules can adhere to different structures on the body surface of carabids. The investigated carabid species differed in the number of hairs, bristles, and dimples on the cuticle [40]. Among the investigated carabid species, *H. signaticornis* is densely punctate and pubescent all over its body, however, *B. lampros* and *B. properans* are nearly hairless with only a few dimples. Furthermore, the diet of the investigated carabid species varies from granivorous like *A. aenea*, or a mixed diet part plant-based and part carnivorous like *H. affinis* or pure carnivore diet consisting of other arthropods like *B. lampros* and or *P. versicolor* [52].

Further studies should aim for a larger sample size to identify underlying patterns in the interaction of carabid beetles and fungi and between fungal species. The previously mentioned propagules’ properties, the diet, the cuticle structure of the carabids should also be paid more attention in subsequent studies as well as the relationship between the beetle-associated fungal population and the fungi in the beetle’s environment.

Nevertheless, carabid beetles do disperse a variety of microorganisms, including fungal species of great economic relevance. This could alter the competitive and antagonistic interactions in the fungal community and affect the growth, the production of primary inoculum, or mycotoxins of economically relevant fungi.

The third hypothesis, regarding the effect of body size and weight on the fungal load, can also be confirmed. Significant differences between the carabid species were detected, regarding endogenous and exogenous total fungi and *Alternaria* DNA, and exogenous propagules of total fungi and *Fusarium*. Overall, larger or heavier species showed a higher fungal load which is corroborated by Yamoah et al. [81]. This trend was confirmed by positive moderate correlation coefficients ranging from 0.43 to 0.58. In contrast, Moyo et al. [5] detected pathogens in similar frequencies in a 3–5 mm large Cocktail Ant (*Crematogaster peringueyi*) than in the 20–45 mm large millipede (*Ommatoiulus moreleti*) but this aspect was not further analyzed by the authors.

In this study, body size and body weight were important factors. However, this only partly explained the distribution of the fungal load between carabid species as the correlation coefficient between fungal load and body size/weight suggests. In the culture-dependent approach, the comparison of the five investigated carabid species showed a relatively clear pattern where the largest species showed the highest fungal load. The morphological structure of the body surface of the carabids was not considered in the analyses but might be the source of the remaining variance. However, morphology only explains a part of the differences since the intra-specific variance and the variance between the morphologically similar species *B. lampros* and *B. properans* are considerably high. Other relevant factors, like the ecology of the carabids, should be investigated too. In the qPCR approach, the midsized species *A. aenea* and *A.* spp. showed a high fungal load, and in the largest species, *P. versicolor*, a large intra-species variance was detected. This suggests that next to the body weight of the beetles, diet is also an important trait. *P. versicolor* feeds on other insects and ingests the mycobiota of its prey, which could explain the great intra-specific variance [52]. Species of the genus *Amara* feed primarily on seeds and grains, which are common hosts for *Fusarium* and *Alternaria* [27,52,82]. This diet probably explains the higher fungal load in this species. Overall, the fungal load is strongly affected by body size and weight. However, the diet and the morphological structure of the body surface of the carabids are relevant as well. Individual differences in the behavior of the insects or the fungal community in the habitat are also affecting the fungal load of the carabid beetles.

Carabids show a remarkable potential to disseminate propagules of different fungal genera, including multiple species of the phytopathogenic *Fusarium* and *Alternaria* fungi.

On the one hand, this dispersal mechanism could enhance crop diseases by transporting propagules from different inoculum sources to the crop plants. Therefore, the dispersal of fungal propagules by ground-dwelling arthropods should be given greater emphasis in the analysis of crop diseases. On the other hand, based on zoochory, carabid beetles could be mobile linkers and alter the fungal community in (semi-) natural habitats and crop fields by exchanging fungal species or strains and link these habitats via their extensive movement pattern. Unraveling the movement behavior of arthropods and their associations with microorganisms is crucial to understand dynamics and patterns in micro-communities.

## Figures and Tables

**Table 1:** List of carabid species, number of individuals (N Indiv.) per species, and measured body size in mm (median with interquartile range, IQR) of carabid beetles screened for fungal propagules by the culture-dependent method.

	Abbreviation	Abbreviation	N	Median
Carabid Species	Long	Short	Indiv.	(IQR)
***Amara aenea***	***A. aenea***	**A. ae**	**4**	**7.52 (1.03)**
*Amara littorea*	*A. littorea*	A. li	1	7.37 (NA)
*Amara ovata*	*A. ovata*	A. ov	1	7.19 (NA)
***Anchomenus dorsalis***	***An. dorsalis***	**An. d**	**5**	**7.47 (0.46)**
***Bembidion lampros***	***B. lampros***	**B. la**	**12**	**3.72 (0.14)**
***Bembidion properans***	***B. properans***	**B. pr**	**5**	**3.79 (0.67)**
***Harpalus affinis***	***H. affinis***	**H. af**	**4**	**9.89 (0.53)**
*Harpalus rufipes*	*H. rufipes*	H. ru	1	15.00 (NA)
*Limodromus assimilis*	*L. assimilis*	L. as	1	10.49 (NA)
*Microlestes minutulus*	*M. minutulus*	M. mi	1	2.80 (NA)
***Poecilus*** **spp.**	***P.*** **spp.**	**P. sp**	**3**	**12.42 (0.41)**

Species in bold were compared with an analysis of variance.

**Table 2:** Number of individuals (Indiv.) of carabid species used for DNA extraction, their body weights in mg (median and interquartile range, IQR) and the number of samples used for DNA extraction, and the average (Ø) number of individuals per sample in the molecular biological approach.

Carabid.	Abbreviation	Abbreviation	Extracted	Weight (mg)	Extracted Samp.
Species	Long	Short	Indiv.	Median (IQR)	(Ø Indiv./Samp.)
*Amara aenea*	*A. aenea*	A. ae	10	11.50 (1.96)	9 (1.11)
*Amara* spp.	*A.* spp.	A. sp	6	11.32 (1.49)	6 (1.0)
*Anchomenus dorsalis*	*An. dorsalis*	An. D	11	5.96 (1.40)	11 (1.0)
*Bembidion properans*	*B. properans*	B. pr	43	1.47 (0.46)	10 (4.3)
*Bembidion tetracolum*	*B. tetracolum*	B. te	30	3.11 (0.80)	13 (2.31)
*Clivina fossor*	*C. fossor*	C. fo	13	3.24 (1.29)	11 (1.18)
*Harpalus affinis*	*H. affinis*	H. af	17	22.62 (3.54)	17 (1.0)
*Harpalus signaticornis*	*H. signaticornis*	H. si	11	5.31 (1.91)	10 (1.1)
*Microlestes minutulus*	*M. minutulus*	M. mi	21	0.49 (0.20)	10 (2.1)
*Poecilus versicolor*	*P. versicolor*	P. ve	16	35.34 (17.33)	16 (1.0)

**Table 3:** Median and interquartile range (IQR) of the DNA concentration (con.) in µg, the DNA quality (A_260/280_) per extracted individual (indiv.) of carabid species, and the number of samples included and excluded for a qPCR analysis in the molecular biological approach. Only samples with an A_260/280_ between 1.6 and 2.1 were included in the qPCR.

Carabid Species	DNA Con. in µg per Extracted Indiv. (Median (IQR)	A_260/280_ per Extracted Indiv. (Median (IQR)	Samples Included in qPCR (Samples Excluded)
*A. aenea*	3152.25 (2269.13)	1.9 (0.04)	9 (0)
*A.* spp.	3513.37 (858.94)	1.87 (0.02)	6 (0)
*An. dorsalis*	2241.00 (924.75)	1.85 (0.04)	10 (1)
*B. properans*	509.63 (438.41)	1.85 (0.11)	7 (3)
*B. tetracolum*	1205.25 (626.63)	1.85 (0.02)	13 (0)
*C. fossor*	518.63 (333.00)	1.83 (0.18)	6 (5)
*H. affinis*	3924.00 (2580.75)	1.87 (0.05)	15 (2)
*H. signaticornis*	2012.63 (1426.5)	184 (0.07)	9 (1)
*M. minutulus*	358.69 (312.47)	1.79 (0.11)	5 (5)
*P. versicolor*	2948.63 (2817.00)	1.87 (0.13)	12 (4)

**Table 4:** Synthesis of the most relevant results.

Criteria	Culture (N = 39)	qPCR (N = 92)
% of Positive Carabids *		
Total fungi	74.4%	100%
*Fusarium*	48.7%	8.7%
*Alternaria*	12.8%	97.8%
Relationships (r_s_) between size/weight and		
Total fungi (range)	0.44	0.47
*Fusarium*	0.43	n.s.
*Alternaria*	n.s.	0.58
Pattern of carabid species-specific differences Large/heavy > small/light	N = 33	N = 92
Total fungi	2 groups, largest species has highest fungal load	3 groups, slightly increased fungal load in smaller and midsized species, large intraspecific variance
*Fusarium*	2 groups, midsized species slightly increased fungal load	N. s., very low frequency,
*Alternaria*	N.s., very low frequency	3 groups, a medium-sized species with very high fungal load

* DNA or propagules detected on or in the carabid beetle with culture-dependent method or qPCR method; n.s. not significant.

**Table 5:** List of *Fusarium (F.)*, *Alternaria* (Al.) species, and their load in a number of CFU, detected on the body surface of 39 carabid beetles. *Fusarium* species were divided into two groups based on their ability to produce macro- and microconidia (Micro) or only macroconidia (Macro). *Alternaria* species were divided into two groups based on the amount produced low or high amounts of mycotoxins.

Fungi Species	Number of CFU	Conidia Type
*F. culmorum*	28.0	Macro
*F. sambucinum*	5.8	Macro
*F. crookwellense*	4.6	Macro
*F. equiseti*	2.4	Macro
*F. oxysporum*	7.0	Micro
*F. arthrosporioides*	4.8	Micro
*F. solani*	4.6	Micro
*F. sporotrichioides*	2.4	Micro
Sum	40.8	Macro
Sum	18.8	Micro
		Toxin amount
*Al. infectoria*	4.6	low
*Al. tenuissima*	2.4	high
*Al. alternata*	1.2	high
*Al. arborescence*	1.2	high
Sum	4.6	Low
Sum	4.8	high

## Data Availability

The data presented in this study are available on request from the corresponding author.

## Supplementary Materials

The following are available online at https://www.mdpi.com/article/10.3390/jof7100863/s1, Figure S1: Photographs of homogenized carabid beetles.

## Abbreviations and Definitions

*Al.*	*Alternaria*
ALT-CFU/beetle	Load of *Alternaria* fungi per carabid beetle
ALTgcn/beetle	*Alternaria* DNA genome copy number per carabid beetle
CFU	Colony Forming Unit
*F.*	*Fusarium*
FUS-CFU/beetle	Load of *Fusarium* fungi per carabid beetle
FUSgcn/beetle	*Fusarium* DNA genome copy number per carabid beetle
gcn	genome copy number
ITS	Internal Transcriber Spacer
ITSgcn/beetle	Total fungal DNA genome copy number per carabid beetle
PDA	Potato dextrose agar
qPCR	real-time polymerase chain reaction
SNA	Synthetic Nutrient-Poor Agar
TOTAL-CFU/beetle	Total fungal number of colony forming units per carabid beetle
Fungal load	number of adhered or ingested fungal propagules or genomes, per individual or sample
Frequency	probability or number of times of the occurrence of a certain fungal genera or species
Abundance	Number of propagules or genomes of fungal genera or species per sample

## Carabid Species

A. ae	*Amara aenea*
A. li	*Amara littorea*
A. ov	*Amara ovata*
A. sp	*Amara* spp.
A.	*Amara*
An.	*Anchomenus*
An. d	*Anchomenus dorsalis*
B.	*Bembidion*
B. la	*Bembidion lampros*
B. pr	*Bembidion properans*
B. te	*Bembidion tetracolum*
C.	*Clivina*
C. fo	*Clivina fossor*
Ca.	*Carabus*
Ca. a	*Carabus auratus*
H.	*Harpalus*
H. af	*Harpalus affinis*
H. ru	*Harpalus rufipes*
H. si	*Harpalus signaticornis*
L.	*Limodromus*
L. as	*Limodromus assimilis*
M.	*Microlestes*
M. mi	*Microlestes minutulus*
P.	*Poecilus*
P. sp	*Poecilus* spp.
P. ve	*Poecilus versicolor*
