# Supplementary material for: Quantifying the Role of Ground Beetles for the Dispersal of Fusarium and Alternaria Fungi in Agricultural Landscapes
Source: J Fungi (Basel). 2021 Oct 14;7(10):863. doi: 10.3390/jof7100863 (PMC8537540; doi:10.3390/jof7100863)
Supplement: Supplementary file 1 [file jof-07-00863-s001.zip › jof-1395174-supplementary.pdf]

Quantifying the Role of Ground Beetles for the Dispersal of *Fusarium* and *Alternaria* Fungi in Agricultural Landscapes

Nadja Heitmann [1,2,*], Michael Glemnitz [1], Peter Lentzsch [1], Ralph Platen [1] and Marina E. H. Müller [1,3]

[1] Leibniz Centre for Agricultural Landscape Research (ZALF), Eberswalder Str. 84, 15374 Müncheberg, Germany; mglemnitz@zalf.de (M.G.); lentzsch@zalf.de (P.L.); ralph.platen@zalf.de (R.P.); mmueller@zalf.de (M.E.H.M.)
[2] Department of Ecology, Brandenburg University of Technology, Cottbus-Senftenberg, Platz der Deutschen Einheit 1, 03046 Cottbus, Germany
[3] Berlin-Brandenburg Institute of Advanced Biodiversity Research (BBIB), Altensteinstr. 34, 14195 Berlin, Germany
* Correspondence: nadja.heitmann@zalf.de or nadja_heitmann@hotmail.de

Supplementary Materials

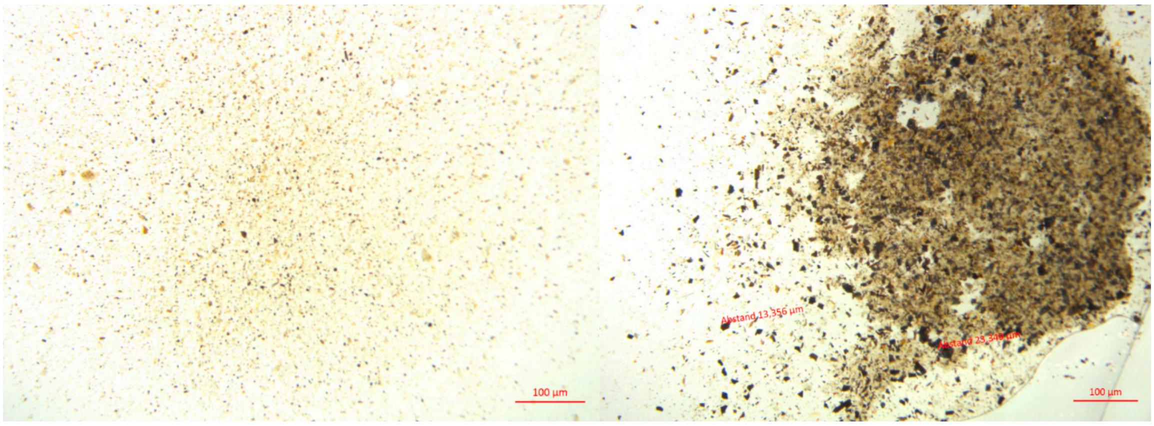

A **B**

Figure S1. Photograph of a homogenized carabid beetle with a Zeiss ZEN 2 Blue Software (Zeiss, Oberkochen, Germany). Homogenization of was performed in 2.0 ml tubes with a high-speed benchtop homogenizer MP FastPrep 24 (MP Biomedicals Germany GmbH, Eschwege, Germany) at a speed setting of 6.5 m/sec and stainless steel grinding beads (MP Biomedicals Germany GmbH, Eschwege, Germany) of a (**A**) *Bembidion properans* for three cycles of 40 sec each and stored for 5 min at −80 °C between the cycles, with two 3.2 mm and one 5.5 mm beads. (**B**) *Poecilus versicolor* for two cycles of 40 sec each and stored for 5 min at −80 °C between the cycles, with two 3.2 mm beads. Based on this preliminary results, for *Harpalus affinis* and *Poecilus versicolor* a third cycle of 40 sec with an additionally 3.2 mm beat was applied for the qPCR method.
